# Calcineurin Inhibitors Suppress Cytokine Production from Memory T Cells and Differentiation of Naïve T Cells into Cytokine-Producing Mature T Cells

**DOI:** 10.1371/journal.pone.0031465

**Published:** 2012-02-16

**Authors:** Kenshiro Tsuda, Keiichi Yamanaka, Hiroshi Kitagawa, Tomoko Akeda, Masanao Naka, Kaori Niwa, Takehisa Nakanishi, Masato Kakeda, Esteban C. Gabazza, Hitoshi Mizutani

**Affiliations:** 1 Department of Dermatology, Mie University, Graduate School of Medicine, Tsu, Mie, Japan; 2 Department of Immunology, Mie University, Graduate School of Medicine, Tsu, Mie, Japan; New York University, United States of America

## Abstract

T cells have been classified as belonging to the Th1 or Th2 subsets according to the production of defining cytokines such as IFN-γ and IL-4. The discovery of the Th17 lineage and regulatory T cells shifted the simple concept of the Th1/Th2 balance into a 4-way mechanistic pathway of local and systemic immunological activity. Clinically, the blockage of cytokine signals or non-specific suppression of cytokine predominance by immunosuppressants is the first-line treatment for inflammatory T cell-mediated disorders. Cyclosporine A (CsA) and Tacrolimus (Tac) are commonly used immunosuppressants for the treatment of autoimmune disease, psoriasis, and atopic disorders. Many studies have shown that these compounds suppress the activation of the calcium-dependent phosphatase calcineurin, thereby inhibiting T-cell activation. Although CsA and Tac are frequently utilized, their pharmacological mechanisms have not yet been fully elucidated.

In the present study, we focused on the effects of CsA and Tac on cytokine secretion from purified human memory CD4^+^T cells and the differentiation of naïve T cells into cytokine-producing memory T cells. CsA or Tac significantly inhibited IFN-γ, IL-4, and IL-17 production from memory T cells. These compounds also inhibited T cell differentiation into the Th1, Th2, and Th17 subsets, even when used at a low concentration. This study provided critical information regarding the clinical efficacies of CsA and Tac as immunosuppressants.

## Introduction

Previously, T cells were classified as Th1 or Th2 subtypes according to the production of defining cytokines such as IFN-γ, IL-12, and IL-4. Several autoimmune diseases, including rheumatoid arthritis (RA) and psoriasis, had been considered to be Th1-cell-mediated disorders driven by a population of T cells producing inflammatory cytokines, such as IL-2, IL-12, TNF-α and interferons [1], [2], [3]. On the other hand, atopic dermatitis (AD) is a Th2-type cytokine-mediated chronic disease associated with increased Th2 cellular infiltration and overproduction of IgE [4]. However, Th1 cells are also involved in AD in both the acute and chronic phases. The discovery of the Th17 lineage and regulatory T cells shifted the simple concept of the Th1/Th2 balance into 4-way system. Th17/22 cells, Foxp3^+^ regulatory T cells (Treg), and IL-10-producing T cells (Tr1) are deeply involved in the mechanisms of the local and systemic immunological milieu [5]. Recently, RA and psoriasis have been characterized as Th17- and Th1-mediated diseases but are mainly Th17-induced disorders. Super Th1 cells are also involved in the development of Th2 disorders [6]. Clinically, the blockage of cytokine signals or non-specific suppression of cytokine predominance by immunosuppressants for T cell-mediated inflammatory disorders is the first line treatment. Cyclosporine A (CsA) is a frequently used immunosuppressant for the treatment of Th1, Th2, and Th17/22 disorders. Many studies have shown that CsA binds to cyclophilin intracellularly to prohibit the synergistic action of Ca2^+^ and suppresses the activation of the calcium-dependent phosphatase calcineurin, thereby affecting the production of cytokines, such as IL-2 and IFN-γ [7], [8]. Tacrolimus (Tac) is also a widely used T-cell-targeted immunosuppressant and a known calcineurin inhibitor. This macrolide from the filamentous bacterium *Streptomyces tsukubaensis* exerts its immunosuppressive effects by inhibiting T-cell activation, particularly in Th1 cells. Several reports have shown that Tac treatment improves psoriasis, which is driven by a mixed population of Th17/Th22 and Th1 cells [9], [10]. However, the pharmacological mechanisms of CsA and Tac have not yet been fully elucidated.

In the present study, we investigated the effects of CsA and Tac on cytokine production from memory CD4^+^ T cells. We also examined whether these compounds influence the differentiation of naïve T cells into Th1, Th2, or Th17 cells.

## Materials and Methods

### Subjects

Ten healthy donors (male/female, 10/0; age, 36±5.1 years) were enrolled in this study. Blood was drawn after obtaining written informed consent from all subjects, and the investigational protocol was approved by the Institutional Review Board (IRB) of Mie University Hospital (Permit Number 2089).

### Antibodies and reagents

Cyclosporine A (CsA) was purchased from Novartis (Basel, Switzerland), and Tacrolimus (Tac) was purchased from Astellas Pharma Inc. (Tokyo, Japan). Phytohemagglutinin (PHA), Phorbol 12-myristate 13-acetate (PMA), and ionomycin were purchased from Sigma-Aldrich (St. Louis, MO, USA). Purified anti-human CD3 mAb, anti-hCD28 mAb, anti-hIFN-γ-PerCP mAb, anti-hIL-4-PerCP mAb, anti-hIL-17-PerCP mAb, and brefeldin A were purchased from BioLegend (San Diego, CA, USA). Anti-hCD4-FITC mAb, anti-hCD45RA-FITC mAb, and anti-hCD45RO-PE mAb were purchased from BD/PharMingen (San Diego, CA, USA). Anti-hIL-4 mAb, anti-hIL-12 mAb, anti- hIFN-γ mAb, and rhIL-12 were purchased from R&D Systems (Minneapolis, MN, USA). Recombinant hIL-1β, rhTGF-β, rhIL-6, and rhIL-2 were purchased from PeproTech (Princeton, NJ, USA). Complete RPMI 1640 medium was made with 10% heat-inactivated fetal bovine serum (FBS, HyClone Laboratories, INC., South Logan, UT, USA), 2.0 mM L-glutamine, 100 U/ml penicillin, and 100 mg/ml streptomycin (Nacalai tesque, Kyoto, JAPAN).

### Purification of CD4^+^ T cells

PBMCs were isolated from fresh heparinized venous blood using Ficoll-Hypaque (Sigma-Aldlich, St. Louis, MO) density gradient centrifugation. Cells were washed twice in phosphate buffered saline (PBS), and purified CD4^+^ T cells were obtained by negative selection using the CD4^+^ T Cell Isolation Kit II (Miltenyi Biotec, Bergisch Gladbach, Germany) according to the manufacturer's instructions. Briefly, for CD4^+^ T-cell selection, PBMCs were incubated for 10 min with 20 µl of the antibody cocktail mixture followed by a 15 min incubation with 20 µl of magnetic beads per 10^7^ cells. Unconjugated CD4^+^ T cells were then isolated from PBMCs by indirect magnetic labeling over MiniMACS separation LS columns. Sorted populations were analyzed by flow cytometry, and the purity of samples ranged between 96 and 99%.

### Separation of naïve and memory CD4^+^ T cells

Naïve CD4^+^ T cells were obtained by positive selection, and memory CD4^+^ T cells were obtained by negative selection from the purified CD4^+^ T cells using the naïve CD4^+^ T Cell Isolation Kit. Untouched memory CD4^+^ T cells passed through the column, whereas magnetically labeled naïve CD4^+^ T cells remained in the column and were washed out with buffer. The purity of both cell populations ranged between 94 and 99%.

### Cell culture of memory CD4^+^ T cells

Memory CD4^+^ T cells, suspended in complete RPMI 1640 culture medium, were plated into a flat-bottomed 24-well plate at 1×10^6^ cells/well and incubated with PMA (1 µg/mL), ionomycin (1 µg/mL) and brefeldin A (1 µg/mL) in the absence or presence of CsA (100 or 1000 ng/mL) or Tac (16.7 or 167 ng/mL) and cultured for 24 h at 37°C with 5% CO_2_.

### Generation of Th1 cells in vitro

Th1 cells were generated by culturing naïve CD4^+^ T cells (1×10^6^/mL) with PHA (1 µg/mL), rhIL-12 (50 ng/mL), and anti-hIL-4 mAb (500 ng/mL) in a flat-bottomed 24-well plate in 1 mL of complete RPMI1640 culture medium at 37°C with 5% CO_2_ in the absence or presence of CsA (100 or 1000 ng/mL) or Tac (16.7 or 167 ng/mL). These stimulated T cells were collected and washed on day 3 and expanded in the same culture medium with the addition of100 U/mL of rhIL-2 for an additional 3 days. On day 6, cells were stimulated with PMA (1 µg/mL) and ionomycin (1 µg/mL) in the presence of brefeldin A (1 µg/mL) for 8 h.

### Generation of Th2 cells in vitro

Th2 cells were generated by culturing naïve CD4^+^ T cells (1×10^6^/mL) with PHA (1 µg/mL), rhIL-4 (200 ng/mL), and anti-hIL-12 mAb (10 µg/mL) in a flat-bottomed 24-well plate in the complete RPMI1640 culture medium at 37°C with 5% CO_2_. Cultures were supplemented with CsA (100 or 1000 ng/mL) or Tac (16.7 or 167 ng/mL). These stimulated T cells were washed on day 3 and expanded in the same culture medium with the addition of 100 U/mL of rhIL-2 for an additional 3 days. On day 6, cells were stimulated with PMA (1 µg/mL), ionomycin (1 µg/mL) and brefeldin A (1 µg/mL). The cells were incubated for additional 8 h.

### Generation of Th17 cells in vitro

Th17 cells were generated by culturing naïve CD4^+^ T cells (1×10^6^/mL) with rhIL-2 (10 U/mL), rhTGF-β (5 ng/mL), rhIL-6 (20 ng/mL), rhIL-1β (10 ng/mL), rhIL-23 (10 ng/mL), anti-hIL-4 mAb (1 µg/mL), anti-hIFN-γ mAb (1 µg/mL), anti-hCD3 mAb (4 µg/mL), and anti-hCD28 mAb (8 µg/mL) in a flat-bottomed 24-well plate in complete RPMI1640 culture medium. Cells were cultured in the absence or presence of CsA (100 or 1000 ng/mL) or Tac (16.7 or 167 ng/mL) at 37°C with 5% CO_2_. On days 3 and 5, the culture plate was centrifuged, and the media was removed and replaced with fresh media containing all cytokines mentioned above, antibodies, and CsA or Tac. On day 6, cells were stimulated with PMA (1 µg/mL), ionomycin (1 µg/mL) and brefeldin A (1 µg/mL). The cells were incubated for 8 h at 37°C in 5% CO_2_.

### Cell surface and intracellular staining of CD4^+^ T cells

Cultured CD4^+^ T cells were collected and washed twice with PBS containing 1% FBS. Cell surface antigens and intracellular cytokines were stained according to the formal Cell Surface Immunofluorescence Staining Protocol and Intracellular Cytokine Staining Protocol (BioLegend). Briefly, for analyzing cytokine production from memory cells, the cells were first stained with anti-hCD4-FITC mAb. To detect cellular differentiation of naïve T cells into cytokine-producing cells, the cells are first stained with anti-hCD45RA-FITC and anti-hCD45RO-PE mAbs. After treatment with the fixation and permeabilization wash buffer, the cells are incubated with PerCP-conjugated anti-hIFN-γ, anti-hIL-4, or IL-17A mAbs. Fluorescence profiles are analyzed by flow cytometry using FACSCalibur (BD Biosciences, San Jose, CA), and the data were analyzed using Cell Quest Pro software (BD Biosciences).

### Statistical analyses

The statistical differences between variables were calculated using the Kruskal-Wallis one-way analysis of variance. P<0.05 was considered to be significant.

## Results

### CsA and Tac inhibit cytokine production from memory CD4^+^ T cells

**Figure 1:**
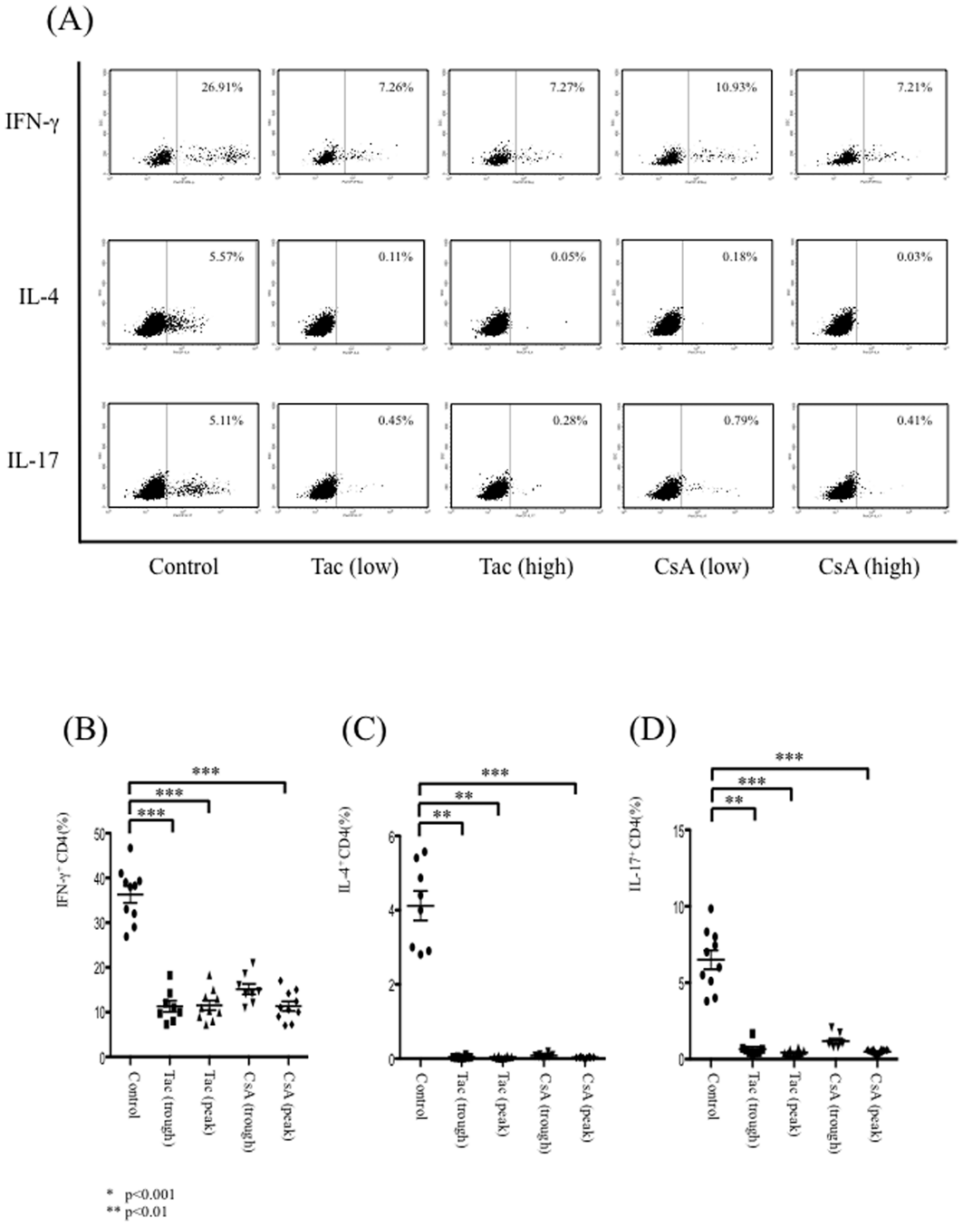
The effects of CsA and Tac on cytokine production from memory CD4^+^ T cells. Memory CD4^+^ T cells were stimulated with PMA and ionomycin in the absence or presence of CsA or Tac. Fluorescence profiles showed distinct cytokine productions from mature cells, and the production of all cytokines investigated here was strikingly suppressed with the addition of CsA or Tac, even at the lower concentration. Representative figures of ten independent experiments are shown (A). The percentages of IFN-γ^+^CD4^+^ T cells (B), IL-4^+^CD4^+^ T cells (C) or IL-17^+^CD4^+^ T cells (D) within the CD4^+^ T cell population were significantly suppressed with the addition of CsA or Tac. Data are expressed as the mean ± SEM.

To determine the impact of CsA and Tac on cytokine production from memory CD4^+^ T cells under distinct stimulation conditions, memory CD4^+^ T cells were stimulated with PMA and ionomycin in the absence or presence of CsA or Tac. Resulting fluorescence profiles showed distinct cytokine production from mature cells, and production of all cytokines investigated here was strikingly suppressed with the addition of CsA or Tac, even at the lower concentration (Fig. 1A). The percentages of IFN-γ^+^CD4^+^ T cells (B), IL-4^+^CD4^+^ T cells (C), or IL-17^+^CD4^+^ T cells (D) were significantly suppressed with the addition of CsA or Tac.

### CsA and Tac inhibit the differentiation of naïve CD4^+^ T cells into cytokine-producing mature cells

**Figure 2:**
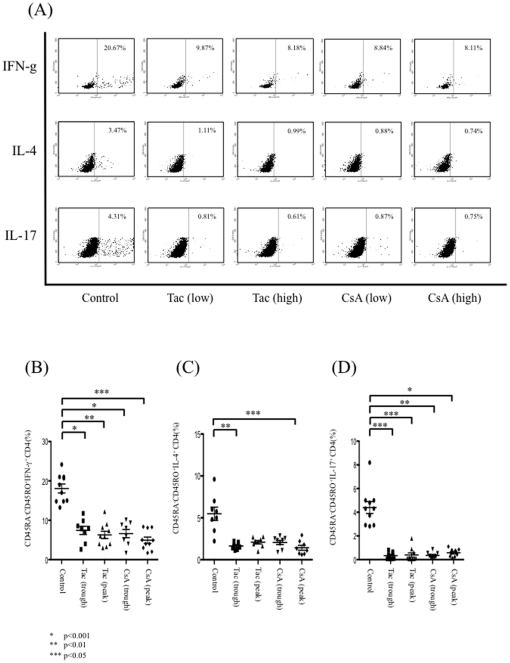
The effects of CsA and Tac on the differentiation of naïve CD4^+^ T cells into cytokine-producing mature cells (Th1/Th2/Th17). Flow cytometric analysis showed abundant cytokine production from CD45RA^−^CD45RO^+^CD4^+^ T cells. Representative figures of ten independent experiments are shown (A). Addition of CsA or Tac lowered the percentage of CD45RA^−^CD45RO^+^IFN-γ^+^ cells significantly compared with the control group (B). The percentages of CD45RA^−^CD45RO^+^IL-4^+^ cells and CD45RA^−^CD45RO^+^IL-17^+^ cells within the CD4^+^ T cell population were also decreased compared with the control group (C,D). Data are expressed as the mean ± SEM.

To investigate the roles of CsA and Tac in the differentiation of naïve CD4^+^ T cells into Th1, Th2, or Th17 cells, naïve CD4^+^ T cells were cultured with the appropriate cytokines and stimulants in the absence or presence of CsA or Tac. Flow cytometric analysis showed abundant cytokine production from CD45RA^−^CD45RO^+^CD4^+^ T cells (Fig. 2A). The addition of CsA or Tac lowered the percentage of CD45RA^−^CD45RO^+^IFN-γ^+^ cells significantly compared with the control group (Fig. 2B). The percentages of CD45RA^−^CD45RO^+^IL-4^+^ cells and CD45RA^−^CD45RO^+^IL-17^+^ cells were also decreased compared with control group (Fig. 2C, D).

## Discussion

CsA and Tac are commonly used to treat immunological diseases; however, the relevant pharmacological mechanisms have yet to be fully understood. In the present study, we focused on the effects of CsA and Tac on the differentiation of naïve CD4^+^ T cells into cytokine-producing memory T cells as well as on the actual cytokine secretion from memory CD4^+^ T cells using T cells from human PBMCs. We used two concentrations of CsA; the low concentration was 100 ng/mL, and the high concentration was 1000 ng/mL; these concentrations are similar to the serum CsA levels at trough and 2-hours peak concentrations of clinically treated patients, respectively. On the other hand, the concentration of Tac was16.7 and 167 ng/mL, and both of these values were derived from safety trough and peak levels for clinical application. We observed successful suppression of cytokine secretion from memory T cells by CsA and Tac. Interestingly, T cell differentiation into the memory cell subset is also prohibited by CsA and Tac even at the lower concentrations.

Previously most autoimmune diseases had been considered to be Th1-cell-mediated; however, recent evidence has indicated that IL-17-producing Th17 cells represent the key effector cells in the induction and development of these disorders [11], [12]. IL-17 has pleiotropic activities, such as coordinating tissue inflammation by inducing the expression of other proinflammatory cytokines, chemokines and matrix metalloproteases [12], [13]. Similar to AD, autoimmune disorders are a complex mixture of Th1 and Th17/22 lineage cells. We analyzed two calcineurin inhibitors that suppressed the production of all examined cytokines.

T cells receive activation signals through T-cell receptors, inducing intracellular Ca^++^ release and activating calcineurin, which dephosphorylates nuclear factor of activated T cells (NFATc). Dephosphorylated NFATc moves into the nucleus and functions as a transcription factor [14]. CsA binding with cyclophilin and Tac coupling with FK-binding protein inhibit calcineurin in T cells. These complexes bind calcineurin, prevent its activation, and block dephosphorylation of NFATc. This lack of dephosphorylation prevents NFATc from activating the transcription of the IL-2 gene. Thus, CsA and Tac reduce activation of IL-2, which is an activator and important growth factor for T cells [15]; therefore, T cell activity is suppressed. CsA and Tac have been clinically used to control Th2-dominant ADs well as Th17/22-dominant psoriasis and related diseases with successful results. Suppression of functional cytokine secretion from differentiated memory T cells by calcineurin inhibitors has been explained through IL-2 gene inhibition. However, the results presented here revealed effects on individual cytokine production from the already differentiated pathogenic T cells in various diseases.

The initial dose of the calcineurin inhibitors for immunological diseases is higher than that of the maintenance dose. The present results confirmed significant effects of calcineurin inhibitors on T cell differentiation at clinically relevant concentrations. Thus, the actions of the calcineurin inhibitors are not limited to non-specific inhibition of T cell-mediated inflammation. These drugs may play fundamental roles in correcting immunological imbalances in allergic and inflammatory diseases. These data also explain the significant and rapid effects of calcineurin inhibitors in atopic dermatitis and related diseases with limited dose and durations.

In conclusion, CsA and Tac inhibit calcineurin in T cells, block the dephosphorylation and translocation of NFATc, inhibit cytokine production from memory CD4^+^ T cells, and prevent the differentiation of naïve CD4^+^ T cells into cytokine-producing memory CD4^+^ T cells. CsA and Tac are promising immunosuppressants for Th1, Th2, and Th17/22 associated disorders.
